# Antidepressant Effects of Intermittent Theta-Burst Stimulation are Associated with Lower Baseline Regional Gray Matter Volumes and Surface Areas in Prefrontal Cortex

**DOI:** 10.1016/j.jpsychires.2025.11.006

**Published:** 2025-11-07

**Authors:** Beatriz Araújo Cavendish, Paulo Suen, Pedro Henrique R. da Silva, Verena Sarrazin, Mariana Baptista, Anne Brito, Matheus Rassi, Lais Razza, Chris Baeken, Jacinta O’Shea, Andre Russowsky Brunoni

**Affiliations:** 1 Service of Interdisciplinary Neuromodulation, Department and Institute of Psychiatry, University of São Paulo Medical School, São Paulo, Brazil; 2 Laboratory of Neuroscience and National Institute of Biomarkers in Psychiatry, Department and Institute of Psychiatry, University of São Paulo Medical School, São Paulo, Brazil; 3 Ghent Experimental Psychiatry (GHEP) lab, Faculty of Medicine and Health Sciences, Department of Head and Skin, Ghent University, Ghent, Belgium; 4 Department of Psychiatry, University Hospital Brussel (UZ Brussel), Brussels, Belgium. Neuroprotection and Neuromodulation Research Group (NEUR), Center for Neurosciences (C4N), Vrije Universiteit Brussel (VUB), Brussels, Belgium; 5 Department of Electrical Engineering, Eindhoven University of Technology, Eindhoven, the Netherlands; 6 Wellcome Centre for Integrative Neuroimaging, Oxford Centre for Human Brain Activity (OHBA), University of Oxford, Department of Psychiatry, Warneford Hospital, Warneford Lane, Oxford, UK; 7 Department of Psychiatry and Peter O’Donnell Brain Institute, UT Southwestern Medical Center, Dallas (TX), USA

**Keywords:** major depressive disorder, intermittent theta-burst stimulation, cortical morphology, gray matter volume, cortical thickness, surface area

## Abstract

**Introduction:**

Major depressive disorder (MDD) is a leading cause of disability worldwide. Intermittent theta-burst stimulation (iTBS) is an effective neuromodulation treatment for MDD, yet treatment response varies and its mechanisms remain unclear. Individual differences in cortical morphology in regions linked to depression may influence treatment outcomes.

**Objective:**

To investigate whether baseline cortical morphology, specifically gray matter volumes (GMV), surface area (SA) and cortical thickness (CT) of the dorsolateral prefrontal cortex (DLPFC) and anterior cingulate cortex (ACC), are predictive to clinical response to a validated iTBS treatment for MDD.

**Methods:**

As part of the BRAEN-MAP trial, fifty-nine patients with MDD underwent daily iTBS sessions targeting the left DLPFC. Depressive symptoms were assessed weekly using the 17-item Hamilton Depression Rating Scale (HDRS-17). Pretreatment structural MRI data were processed with FreeSurfer to extract GMV, SA, and CT from bilateral DLPFC and ACC. Associations with symptom improvement were tested using mixed linear models.

**Results:**

Fifty patients (mean age = 39.98 years, SD=10.79; 84.6% female) were included in the analysis. HDRS-17 scores decreased from 18.23 (SD=2.67) to 8.0 (SD=5.1) after six weeks. Smaller baseline GMV in the left DLPFC (area p9-46v) and right pregenual ACC (area s32), as well as reduced SA in bilateral DLPFC (p9-46v, 8C, a9-46v, 46, 8Av, i6.8) and ACC subregions (s32, d32, 25, a32pr), were significantly associated with greater symptom improvement. No associations were found for CT.

**Conclusion:**

A significant association between individual cortical morphology and depression improvement after iTBS was seen, showing that variations in pretreatment cortical structure may influence neuromodulation treatment efficacy.

## Introduction

Major depressive disorder (MDD) is a highly prevalent and debilitating mental disorder, with substantial rates of resistance to antidepressant treatments (Kessler et al., 2003); (Rush et al., 2006). Non-invasive brain stimulation (NIBS) techniques such as intermittent theta-burst stimulation (iTBS), a form of repetitive transcranial magnetic stimulation (rTMS) (Blumberger et al., 2018), has emerged as a safe and effective alternative as depressive treatment (Brunoni, Chaimani, et al., 2017).

Current models conceptualize iTBS as a network-based therapy (Tik et al., 2023), wherein the magnetic pulses delivered to a single brain region, most commonly the dorsolateral prefrontal cortex (DLPFC) due to its central role in emotion regulation (Golkar et al., 2012), modulate the activity and connectivity of areas engaged with the stimulation site (Castrillon et al., 2020; Eshel et al., 2020). This repeated modulation changes brain activity patterns locally and across networks, ultimately leading to a reduction in depressive symptoms (Eshel et al., 2020). For instance, the stimulation of the DLPFC via rTMS was shown to change its functional connectivity with the anterior cingulate cortex (ACC) through a specific meso-cortico-limbic network (Tik et al., 2017) and to restore abnormal ACC connectivity within the default mode network (DMN) (Liston et al., 2014). Notably, the degree of baseline resting-state functional anticorrelation between the DLPFC and the subgenual ACC is a robust biomarker for rTMS response (Cash et al., 2019; Fox et al., 2012), further supporting the role of these regions in the mechanisms of rTMS for depression. Despite significant advances in rTMS protocols, substantial inter- and intra-individual variability in clinical outcomes remains.

One potential contributing factor is the cortical morphology of the stimulation targets. For instance, a thinner baseline cortical thickness (CT) of the rostral part of the ACC was associated with a better clinical response to a 10 Hz rTMS protocol, and a post-treatment increase in CT of the same region was similarly associated with improved clinical outcomes (Boes et al., 2018). In another study, the typical reductions in prefrontal gray matter volume (GMV) seen in depression were reversed following a daily 10 Hz rTMS protocol administered over five weeks. Patients who exhibited greater antidepressant effects also showed increased GMVs in prefrontal regions such as the left medial frontal gyrus, ACC, and medial orbital after treatment, suggesting that the intervention promotes a neuroplastic response linked to morphology (Lan et al., 2016). However, contrasting results from a trial with an accelerated iTBS protocol over four days with five daily sessions indicated that greater CT in the right caudal ACC predicted a better clinical response (Baeken et al., 2021). Despite the heterogeneity of stimulation protocols, the influence of different morphological variables suggests an interaction between these factors rather than a clear understanding of the underlying mechanisms. Therefore, a biomarker to predict clinical improvement following TMS has yet to be established.

Given that distinct morphometric measures, namely GMV, CT, and surface area (SA) reflect partially different neurobiological substrates (Panizzon et al., 2009; Winkler et al., 2018) and might provide complementary information about cortical architecture and plasticity (Winkler et al., 2012), examining these features together may offer a more comprehensive understanding of the structural variables associated with treatment response. While most previous studies have focused on a single morphometric measure, we aimed to determine whether these metrics are differentially associated with clinical outcomes following iTBS, thereby disentangling their individual predictive value and contributing to a more integrative view of neuromodulation mechanisms.

Therefore, the primary goal of this study was to investigate the association between anatomical baseline characteristics of the DLPFC and ACC focusing on GMV, CT, and SA, and depression improvement in an open-label iTBS trial, the Brazil-England Study on Mechanisms and Response Predictors of iTBS Treatment (BRAEN-MAP). Based on prior evidence linking brain structure to neuromodulation response through neuroplastic changes in target regions, we hypothesized that baseline cortical morphology of the DLPFC and ACC would predict antidepressant response. The regions of interest were selected a priori based on their previously reported association with neuromodulation outcomes (Baeken et al., 2021; Boes et al., 2018; Bulubas et al., 2019), their well-established role in the pathophysiology of MDD (Fonseka et al., 2018), and their frequent targeting in neuromodulation protocols (Berlim et al., 2014).

## Materials and Methods

### Overview

The BRAEN-MAP trial was conducted between August 2021 and October 2022 at the Institute of Psychiatry, Hospital das Clínicas, University of São Paulo Medical School, Brazil, where all recruitment and data collection took place. The study was registered on ClinicalTrials.gov (NCT04969549) and approved by local and national ethics committees (CAAE: 52384921.6.0000.0068). Participants were recruited via social media and internal hospital communications. After a prescreening phase with online questionnaires, they underwent an online individual evaluation, performed by a certified psychiatrist, for eligibility criteria assessment. Data collection was managed using the Research Electronic Data Capture (REDCap) platform (Harris et al., 2009). The treatment protocol consisted of 20 daily iTBS sessions on workdays, with an MRI exam performed at baseline, before the first stimulation session. Weekly clinical evaluations were conducted using the 17-item Hamilton Depression Rating Scale (HDRS-17) (Hamilton, 1960). After completing the treatment phase of four weeks, participants returned for a final visit two weeks later, which was designated as the endpoint in our analysis.

### Participants

Participants were eligible for inclusion if they were aged 18 to 65 years, had a HDRS-17 score greater than 14, and met the criteria for MDD according to the Diagnostic and Statistical Manual of Mental Disorders (DSM-5), as assessed by the Mini International Neuropsychiatric Interview (MINI)(Amorim, 2000). Exclusion criteria were the presence of comorbid mental disorders such as substance dependence and abuse, bipolar disorder, schizophrenia, dementia, obsessive-compulsive disorder, post-traumatic stress disorder, severe neurological or medical conditions, and psychotic or manic symptoms. The only psychiatric comorbidities allowed were the anxiety related ones, such as generalized anxiety disorder, panic disorder specific and social phobias. Participants with contraindications to iTBS or MRI (e.g., metal implants in the head, claustrophobia) were excluded. Antidepressant or psychotropic medication doses were required to remain stable for at least six weeks prior to the study and should not be changed during the trial. Also, benzodiazepine use was limited to a maximum of 20 mg/day diazepam-equivalent. Significant worsening of clinical symptoms, such as an HDRS-17>28 or psychosis, resulted in discontinuation from the study and an immediate psychiatric referral.

### Interventions

The iTBS sessions were administered between 9am and 3pm, with all participants undergoing an MRI exam before the first treatment session. The resting motor threshold (rMT) was visually determined in the first two days of each treatment week. If the rMT differed by more than five percentage points between these days, reassessment was performed on subsequent days until there was no significant variation from one day to the next. The rMT was defined as the minimum intensity needed to elicit a visible contraction in the first dorsal interosseous muscle in three out of five TMS pulses delivered over the motor cortex hotspot (Groppa et al., 2012).

**Fig. 1:**
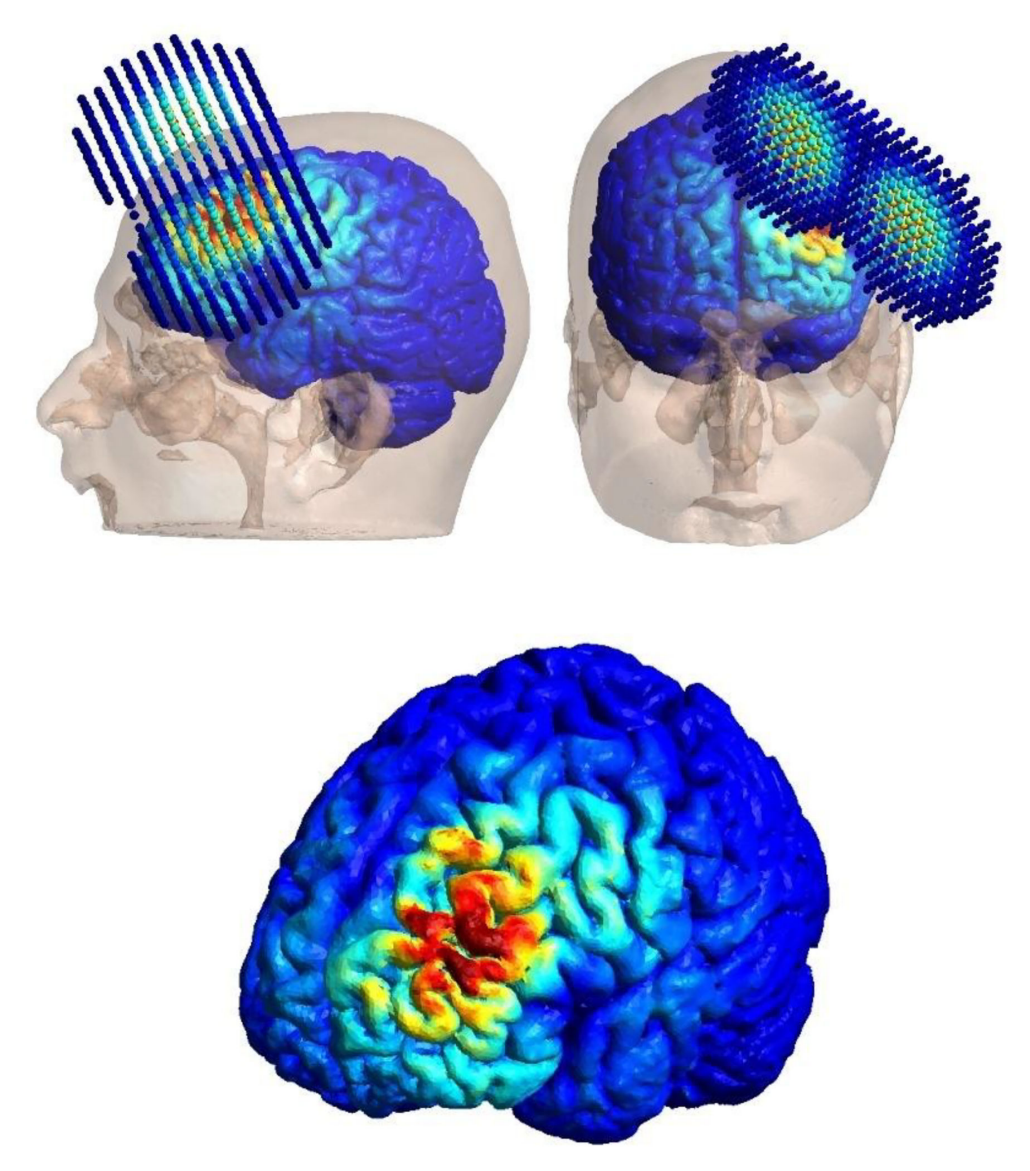
Coil positioning and electric field strength beneath the coil, centered over F3 as determined by the modified Beam F3 method. Modeling parameters are detailed in the supplementary material.

Sessions were performed using a MagPro X100 TMS device and a figure-of-8 B65 coil (MagVenture, Farum, Denmark). The coil was positioned over the left DLPFC following the modified Beam F3 method (Mir-Moghtadaei et al., 2015), angled 45 degrees relative to the midline (see Fig. 1 for an illustration of the electric field beneath the coil). Electric-field simulations were conducted to visualize the positioning and magnitude of the induced field under the stimulation parameters (Fig. 1), providing a spatial reference for the cortical regions influenced by stimulation and enabling direct comparison between field intensity distribution and the areas identified as predictive of clinical response. Each session consisted of 50 Hz triple bursts delivered at 5 Hz, repeated every two seconds, interspersed with eight seconds of rest. Based on studies showing that higher daily pulse doses may enhance antidepressant effects without compromising tolerability (Cole et al., 2020; Li et al., 2014, 2020; Plewnia et al., 2014), a high-dose iTBS protocol of 1,800 pulses per session was administered. This approach has been increasingly adopted in iTBS trials and maintains a brief session duration, supporting feasibility in clinical settings. Each session lasted 9 minutes and 24 seconds, with 1,800 pulses per session. Stimulation intensity was individualized for each participant at 100% of their rMT.

### MRI acquisition

The MRI exams were performed using a 3T machine (Achieva Philips) located in the Instituto de Radiologia, at the Hospital das Clínicas da Universidade de São Paulo. T1-weighted data was acquired with the following parameters: TR = 7.0 ms, TE = 3.2ms, excitation angle = 8°, direction 1.5, FOV = 240 x 240 mm^2^, matrix = 240 x 240 pixels, 180 slices of 1 mm each, without gap, with resulting voxel size of 1x1x1 mm^3^. T2-weighted sequences were acquired using the TR = 2100 ms, TE = 287 ms, 180 axial slices (thickness = 1mm), FA = 90º, FOV = 256 x 256 mm^2^.

### MRI data processing and ROI extraction

We used FreeSurfer brain imaging software (http://surfer.nmr.mgh.harvard.edu/, version 7.2.0) (Fischl, 2012) to automatically process all participants’ T1- and T2-weighted images using the standard ‘recon-all’ pipeline. This pipeline begins by registering the structural images to the Talairach atlas (Collins et al., 1994) and performing intensity normalization to correct for scanner inhomogeneities. Non-brain tissue is removed during the skull-stripping step (Ségonne et al., 2004). The brain is then aligned to the Talairach atlas through an initial transformation, which is subsequently refined for greater precision. Next, white matter segmentation is performed to identify and label white matter regions, followed by the reconstruction of white matter and pial surfaces for each hemisphere (Fischl et al., 2002). The cortical surface is inflated and mapped onto a spherical template for labeling according to Desikan-Killiany (Desikan et al., 2006) and Destrieux atlas (Destrieux et al., 2010). The cortical ribbon, which delineates the boundary between white and gray matter, is further refined. Individual cortical surfaces are registered to a common spherical template, enabling cross-subject comparisons.

The cortical morphological variables examined in this study were estimated based on a surface-based stream, which maintains consistent topology between white and pial surfaces, with each vertex on the white surface having a corresponding pair on the pial surface (Van Essen et al., 1998). CT at each vertex was estimated as the average of (1) the distance from each white surface vertex to its closest point on the pial surface, and (2) the distance from each pial surface vertex to its closest point on the white surface (Fischl & Dale, 2000). The SA was computed as the interface between gray and white matter (Winkler et al., 2012), and GMV was calculated as the product of SA and CT at each vertex. The outputs were inspected for segmentation errors, and total intracranial volume (TIV) was calculated to account for individual differences in brain size.

We used the multi-modal cortical parcellation scheme (HCP-MMP1.0) based on data from the Human Connectome Project (Glasser et al., 2016) to extract regional GMV, CT and SA for subsequent analyses. The HCP-MMP1.0 is a comprehensive cortical-brain parcellation that combines features derived from resting state functional MRI, task fMRI, myelin maps, and CT data. In this parcellation, the DLPFC and ACC are subdivided into smaller areas based on their distinct functional and structural features. The DLPFC consists of 13 subregions per hemisphere, labeled as: 46, SFL, 8Ad, 8C, 8BL, 8Av, 9p, 9a, p9-46v, a9-46v, i6-8, s6-8, and 9-46d. The ACC includes 11 subregions per hemisphere: s32, a24, and p32 as composing the pregenual ACC (pACC), 25 composing the subgenual ACC (sgACC), and a32pr and 33pr composing the supracallosal ACC (ACCsup). Our in-house scripts first used the “mri_surf2surf” command to align the fsaverage atlas to each participant’s native space. Next, we applied the “mris_anatomical_stats” command to extract GMV, SA and CT statistics for all regions of the atlas, and “aparcstats2table” to aggregate regional statistics into a single table. Finally, all the data were exported to R Studio for statistical analyses.

### Statistical analyses

The BRAEN-MAP trial was originally powered to detect early functional changes in amygdala reactivity in association with reduced negative cognitive bias, which constituted its primary aim. The calculation was informed by prior work with SSRIs (Godlewska et al. 2012) and adapted to examine whether similar effects could be elicited by iTBS. The sample size of 50 participants was also considered adequate for the present analyses, as it is consistent with previous neuroimaging trials with comparable designs and objectives (Boes et al. 2018; Baeken et al. 2021; Lan et al. 2016).

Analyses were conducted in R Studio (version 4.4.2) (Team, 2020). Linear mixed-effect models (LMMs) were performed to evaluate the association between baseline GMV, CT, and SA and symptom reduction over the treatment course. GMV values were normalized by the total intracranial volume (TIV) to account for differences in brain size. The dependent variable was depression severity which was weekly assessed using HDRS-17 across the treatment period. Time (baseline and weeks 1–6), baseline GMV, CT, SA and their interaction were included as fixed effects in the models, and a random intercept for each participant accounted for within-subject variability. Age and sex were included as covariates to control for their potential effects. Each subdivision of the DLPFC and ACC, defined by the HCP-MMP1.0 parcellation, was analyzed in a separate model. Also, each morphometric measure (GMV, CT, and SA) was entered into separate models to assess its independent association with clinical response and to avoid possible collinearity effects. The false discovery rate (FDR) method was used to correct for multiple comparisons (Benjamini & Hochberg, 1995). Results were considered significant at a p-FDR threshold of 0.05. Marginal R-squared was used as the effect size.

## Results

### Participants

Out of 296 screened volunteers, 59 were enrolled and 55 completed the trial. Withdrawals occurred due to complications from other clinical conditions (n=1), symptom worsening (n=1), lack of treatment response (n=1), and work-related reasons (n=1). Among the completers, two participants were excluded from the analyses due to image artifacts, and three were excluded for missing weekly clinical evaluations. As a result, 50 participants who completed both the baseline MRI and all clinical assessments were included in the final analysis. Their demographic and clinical characteristics are summarized in Table 1.

### Symptom Severity Improvement

**Fig. 2:**
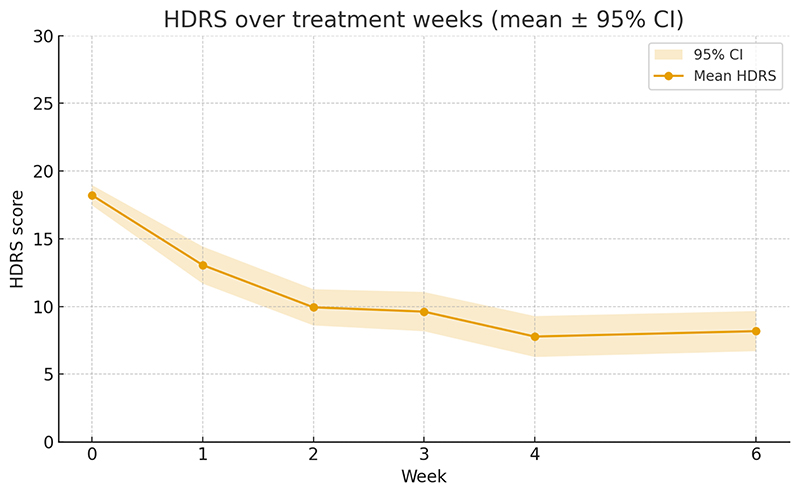
Change in 17-Item Depression Rating Scale (HDRS-17) scores over treatment. The shaded area around the line represents the 95% confidence interval.

There was a significant effect of time on HDRS-17 scores, indicating a progressive reduction in symptom severity from week 1 to week 6. Compared to baseline, symptom reductions were significant at all time points (week 1–6, all *p*s<0.001) (Fig. 2).

### Gray Matter Volumes

**Fig. 3:**
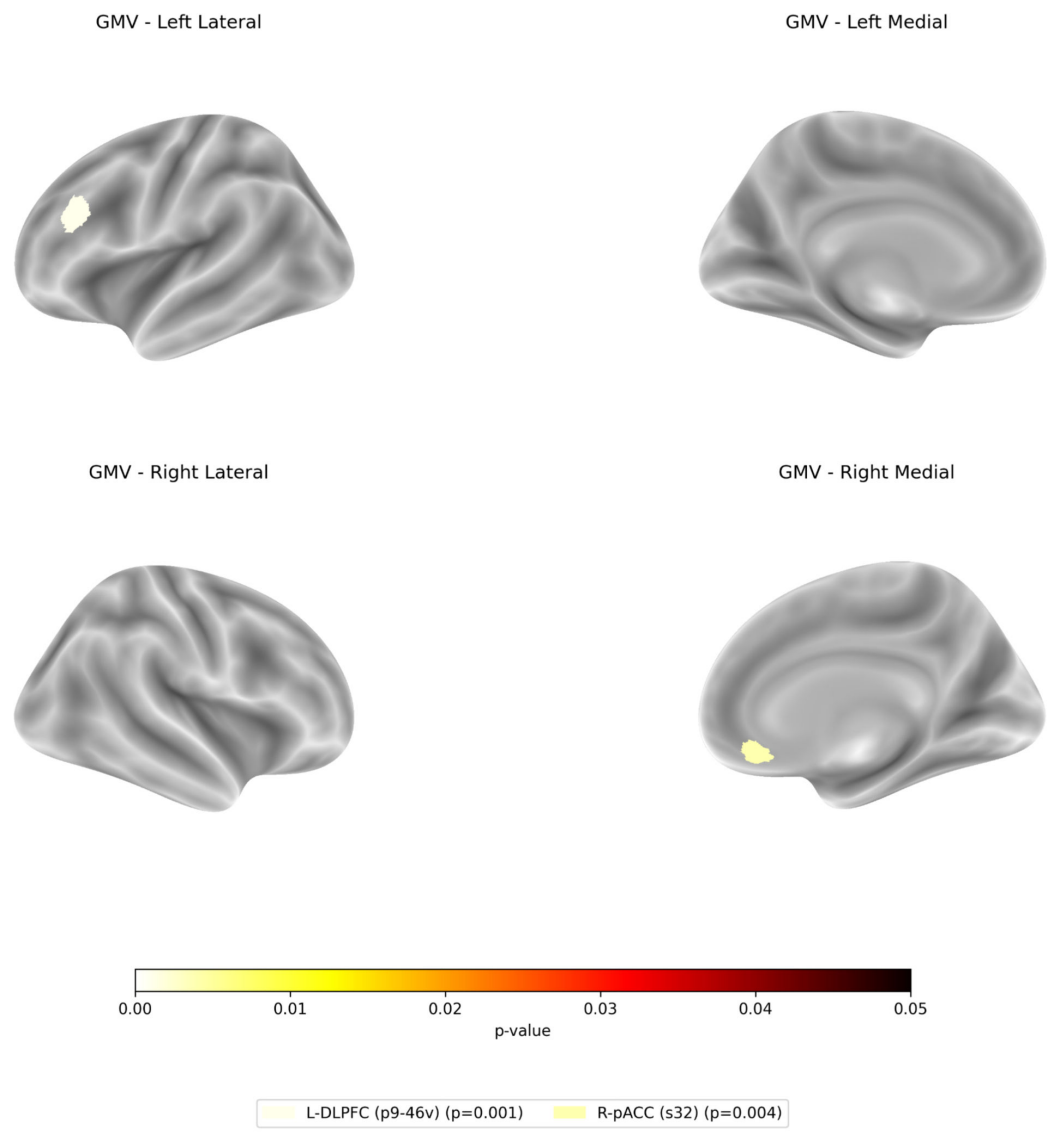
Regions showing significant associations between baseline GMV and symptom improvement from baseline to week 6. Lower volumes in the left DLPFC and right pACC were associated with greater clinical improvement following treatment. Reported p-values are FDR-corrected.

The lower baseline GMV in the left DLPFC (p9-46v) and right pACC (s32) the larger depression symptom improvement (t(248) = 4.27, p-FDR < 0.001, R^**2**^ = 0.29; t(248) = 3.19, p-FDR = .004, R^**2**^ = 0.27, respectively) (Fig. 3 and Table 2).

### Cortical Thickness

We found no significant association between baseline CT and changes in symptom severity across time (all *p*s-FDR > 0.05).

### Surface Area

More reduced baseline SA in the left DLPFC was significantly associated with symptom improvement (p9.46v: t(248) = 4.06, p-FDR < 0.001, R^**2**^ = 0.28; 8C: t(248) = 3.67, p-FDR < 0.001, R^**2**^ = 0.27; a9.46v: t(248) = 2.47, p-FDR = 0.038, R^**2**^ = 0.26; 46: t(248) = 3.06, p-FDR = 0.006, R^**2**^ = 0.26). In the right DLPFC, a stronger reduction baseline SA in 8Av and i6.8 were also significantly associated with changes in depression severity (t(248) = 3.14, p-FDR = 0.001, R^**2**^ = 0.27; t(248) = 2.51, p-FDR = 0.001,R^**2**^ = 0.25).

**Fig. 4:**
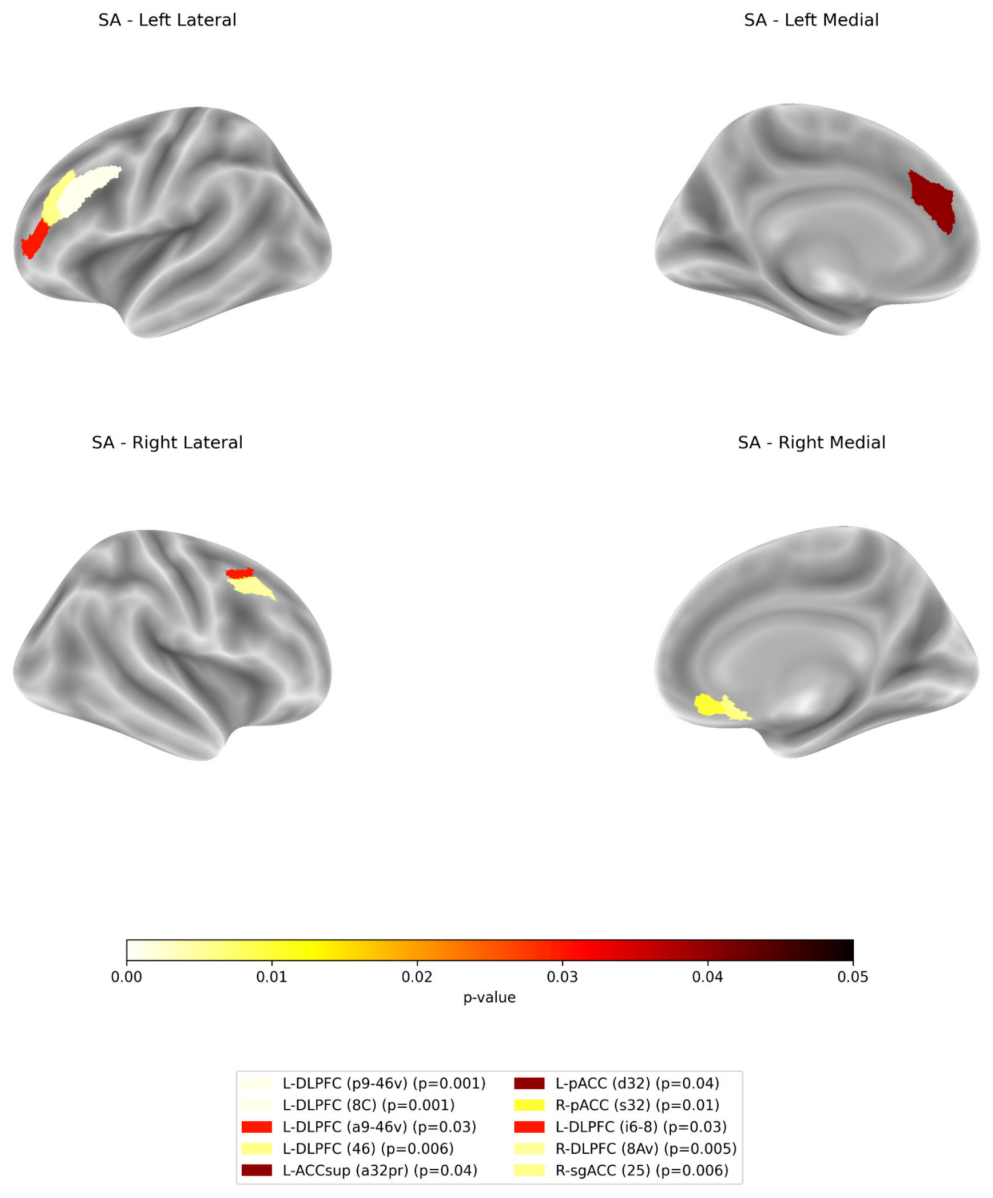
Regions showing significant associations between baseline SA and symptom improvement from baseline to week 6. Reduced SA in the bilateral pregenual, right subgenual, and left supracallosal ACC, as well as bilateral DLPFC, were associated with greater clinical improvement following treatment. Reported p-values are FDR-corrected.

Similarly, a stronger reduction in baseline SA in the right ACC was significantly associated with symptom improvement (sgACC area 25: t(248) = 3.07, p-FDR = 0.006, R^**2**^ = 0.27; pACC area s32: t(248) = 2.93, p-FDR = 0.01, R^**2**^ = 0.26, and area d32: t(248) = 2.42, p-FDR = 0.01, R^**2**^ = 0.27), as well as left ACCsup (a32pr: t(248) = 2.43, p-FDR = 0.04, R^**2**^ = 0.26). Results are shown in Fig. 4 and Table 2.

## Discussion

We examined whether cortical morphology of key brain regions linked to depression were associated with symptom improvement following a treatment regimen consisting of 20 daily iTBS sessions targeting the left DLPFC. We found that a lower baseline gray matter volume in a subregion of the left DLPFC and right pACC was associated with greater antidepressant response. Similarly, smaller SA in bilateral DLPFC subregions, right sgACC and pACC, and left ACCsup was also linked to better clinical outcomes. No significant associations were found for cortical thickness. These findings support the notion that individual differences in cortical morphology may influence the efficacy of neuromodulation therapies, potentially affecting how currents propagate within the targeted sites and their networks (Filmer et al., 2019).

Depression has consistently been associated with structural and functional reductions in the ACC and DLPFC (Pizzagalli, 2011). Longitudinal studies indicate progressive volume decline in these regions among patients with treatment resistant depression (Frodl et al., 2008). Lower rostral ACC volume and functional activity have been linked to unfavorable treatment response, illness chronicity, and higher hospitalization rates (Costafreda et al., 2009; Fu et al., 2013), while lower DLPFC gray matter volume correlates with greater symptom burden, cognitive dysfunction, and reduced pharmacological efficacy (Li et al., 2010; Leung et al., 2009). In this context, our findings can be interpreted in light of the neuroplastic mechanisms thought to underlie neuromodulation therapies, particularly long-term potentiation (LTP) and long-term depression (LTD) (Huang et al. 2005). Although lower gray matter volumes in prefrontal regions have been associated with more severe and treatment-resistant depression, such structural characteristics may not necessarily represent a disadvantage for neuromodulation therapies. Instead, they might indicate greater potential for neuroplastic remodeling at target sites, as stimulation-induced processes can preferentially act on regions with more “room” for plastic change. From this perspective, reduced baseline morphometry could reflect a cortex that remains responsive to neuromodulation induced plasticity, facilitating volumetric increases or microstructural reorganization in response to stimulation. Supporting this view, longitudinal neuroimaging studies have demonstrated increases in cortical volume following rTMS treatment in patients with depression (Lan et al., 2016), consistent with the engagement of structural plasticity mechanisms. Preclinical evidence further supports this interpretation: neurostimulation paradigms enhance neurogenesis, synaptogenesis, and dendritic remodeling, while pharmacological inhibition of these processes prevents antidepressant effects (Perera et al., 2007; Schloesser et al., 2015). In humans, structural MRI studies have shown that individuals with lower baseline volume in the DLPFC or ACC sometimes exhibit greater clinical improvement following TMS (Boes et al., 2018; Baeken et al., 2017), consistent with a higher capacity for stimulation-induced remodeling.

Furthermore, structural differences in the brain may reflect underlying variability in both structural and functional connectivity, which could influence how effectively these regions interact with and modulate depression-related brain circuits (Pizzagalli, 2011). This latter hypothesis is supported by previous findings showing that structural connectivity between patient-specific stimulation targets in the DLPFC and the caudal and posterior portions of the ACC hold potential in predicting clinical response to accelerated iTBS for depression (Klooster et al., 2020). Similarly, baseline fronto-insular effective connectivity and salience network connectivity have been associated with immediate clinical response to rTMS (Iwabuchi et al., 2019). Together, these findings underscore the role of morphological characteristics and structural connectivity profiles of stimulation targets and their networks in shaping rTMS outcomes.

Unlike previous studies that focused on a single morphological feature, such as cortical thickness or gray matter volume, we conducted a comprehensive analysis of three baseline morphological measures within the same cohort to evaluate their association with iTBS clinical outcomes. Importantly, they reflect distinct structural properties. Cortical thickness is the distance between the white matter boundary and the pial surface, potentially linked to neuronal density and glial support (Dahnke et al., 2013). Surface area refers to the total unfolded area of the cortical sheet and is relatively stable over time, and gray matter volume combines thickness and surface area, capturing variations in both (Panizzon et al., 2009). Interestingly, some of the regions showing structural associations with symptom improvement, namely the right subgenual and pregenual ACC and left DLPFC area 46, overlap with areas previously identified as connectivity-based predictors of treatment response (Fox et al., 2012), suggesting a potential link between brain structure and function in the context of biomarkers. Recent protocols have indexed these regions as the most optimized targets for rTMS in depression treatment, using as a stimulation site the individual regions showing the highest anticorrelation between them, given its robust association with clinical efficacy (Cash et al., 2021; Fox et al., 2012; Weigand et al., 2018). Ultimately, if a reliable correlation between structural and functional biomarkers can be established, structural biomarkers would have the advantage of requiring less analytical complexity. However, further research in larger cohorts is needed to replicate this finding and achieve the external validation necessary for clinical application.

While gray matter volume has been more consistently associated with rTMS treatment response, evidence linking SA to neuromodulation outcomes remains scarce. SA captures the tangential expansion of the cortex and reflects the number and spatial organization of cortical columns, which in turn shape local circuit complexity and long-range connectivity (Rakic, 2009). These structural properties are relevant for neuromodulation because stimulation effects seem to depend not only on synaptic density but also on the biophysical geometry of targeted neural populations. Computational models have shown that neuron morphology directly influences susceptibility to externally induced electric fields, with larger dendritic fields and axonal projections showing increased polarization during stimulation (Radman et al. 2009; Aberra et al. 2020). Supporting this view, intracellular recordings in human cortical tissue reveal substantial variability in excitability across pyramidal neurons (Moradi Chameh et al. 2021), which suggests that regional cytoarchitectural complexity could modulate stimulation effects. Thus, SA may reflect a cortical architecture characterized by greater cellular diversity, broader connectivity, and enhanced capacity for activity-dependent plasticity, underscoring its relevance as a measure of interest for neuromodulation studies.

Previous studies have reported mixed results regarding the association between cortical morphology and neuromodulation outcomes. One study, for instance, found that changes in cortical thickness in the left rostral ACC following rTMS were correlated with clinical response, with patients who exhibited greater symptom improvement showing increased thickness in this region, whereas those with less improvement showing cortical thinning (Boes et al., 2018). Another study found that higher baseline cortical thickness in the right caudal ACC predicted better clinical response to accelerated iTBS, with post-treatment changes in this region also correlating with symptom improvement (Baeken et al., 2021). Although the findings are contrasting, it is important to consider that they used different stimulation protocols (rTMS vs. iTBS) and treatment regimens, with one conducting once-daily sessions, and the other an accelerated protocol over a shorter period of time, which might activate distinct neural mechanisms. Regarding gray matter volume, one study reported a significant increase in volume of clusters of brain regions following five-week rTMS treatment, with a particular increase in volume in the ACC showing a correlation with greater clinical improvement (Lan et al., 2016). The morphology-response relationship has also been investigated with other types of neuromodulation, such as transcranial direct current stimulation (tDCS). Interestingly, one study aiming to compare neuroimaging effects of tDCS and pharmacotherapy found that higher gray matter volume in the prefrontal cortex was associated with a greater antidepressant response to tDCS, and this effect was not seen in patients receiving selective serotonin reuptake inhibitors or placebo (Brunoni, Moffa, et al., 2017). These results indicate that the brain structural characteristics of neuromodulation targets may be particularly relevant for treatments that directly engage these areas, in contrast to pharmacological approaches that exert their effects more diffusely. In the case of electroconvulsive therapy (ECT), there is robust evidence linking brain morphological changes to treatment response. For instance, previous studies demonstrated that lower volumes of specific subparts of the hippocampus were associated with better outcomes of ECT (Cao et al., 2018). Increases in gray matter volume following ECT have been consistently observed in regions such as the caudate nucleus, medial temporal lobe, insula, and posterior superior temporal areas (Bouckaert et al., 2016; Gbyl & Videbech, 2018; Sackeim, 2020; Sartorius et al., 2016). Longitudinal ECT studies using neuroimaging have reported both localized and widespread volumetric changes across several brain regions (Abbott et al., 2014; Ousdal et al., 2020), suggesting that structural plasticity may represent a key mechanism underlying the clinical efficacy of ECT.

Finally, another possible and not mutually exclusive hypothesis is that lower baseline volume and surface area may enhance treatment response via an interaction between the structural characteristics of these regions and the local electric field induced by stimulation. It is plausible that reduced cortical volume may lead to greater electric-field engagement in these regions, further enhancing the therapeutic effect. In the context of ECT, significant interactions between EF strength and clinical response have been observed (Argyelan et al., 2019), suggesting that anatomical features may influence how stimulation impacts neural circuits. This may extend to TMS as well, but further research is needed to clarify whether such an association exists for TMS and, if so, to what extent it contributes to treatment efficacy.

### Limitations

Although we achieved a considerable sample size, an important limitation is the absence of a control group to rule out the possibility that the observed antidepressant response may be driven by unknown factors underlying clinical improvement. Additionally, although sex and age were included as covariates in all statistical models, the predominance of female participants (approximately 85%) may limit the generalizability of the findings. Nonetheless, this distribution reflects the higher prevalence of major depressive disorder among women reported worldwide (Kuehner, 2017; Salk et al., 2017). This study employed a non-neuronavigated iTBS protocol, and the use of neuronavigation could enhance the precision of targeting, increasing confidence that stimulation reaches the intended site, whose morphology, in turn, may be associated with treatment response. Finally, future studies incorporating MRI at multiple timepoints may offer a broader framework for assessing longitudinal changes and their relationship with symptom improvement, potentially yielding more robust conclusions regarding the role of cortical morphology in the mechanisms of iTBS.

## Conclusions

This study identifies potential neuroimaging biomarkers of iTBS response in patients with major depressive disorder. The observed associations between clinical efficacy and morphological characteristics of the DLPFC and ACC underscore the relevance of these regions in the therapeutic mechanisms of iTBS. Specifically, lower gray matter volumes and surface areas in these areas were linked to greater antidepressant response, contributing to the ongoing search for predictors of neuromodulation treatment outcomes.

## Supplementary Material

Supplementary Material

## Figures and Tables

**Table 1:** Demographic and Clinical Characteristics of the sample.

Variable	N=50
Mean age (SD)	39.98 (10.79)
Gender (F), No. (%)	44 (84.6%)
Marital status (Married), No. (%)	13 (25%)
Ethnicity (White), No. (%)	30 (57.7%)
*HDRS-17 scores at*	
Baseline, mean (SD)	18.23 (2.67)
Week 1, mean (SD)	13.15 (4.87)
Week 2, mean (SD)	10.15 (4.91)
Week 3, mean (SD)	9.39 (5.03)
Week 4, mean (SD)	7.23 (5.06)
Week 6, mean (SD)	8 (5.1)

*Abbreviation* HDRS-17: Hamilton Depression Rating Scale, 17-item version. *Note:* Values are presented as mean and standard deviation (SD) for continuous variables and percentages for categorical variables.

**Table 2:** Significant interactions between time, symptom improvement and cortical morphology (GMV and SA).

Variable	ROI		Coefficient	SE	Statistic	p-FDR	R^2^
SA	L-DLPFC	p9.46v	0.49	0.12	4.01	<0.001	0.28
		8C	0.44	0.12	3.67	<0.001	0.27
		46	0.37	0.12	3.06	0.006	0.26
		a9.46v	0.30	0.12	2.47	0.03	0.26
	R-DLPFC	i6.8	0.31	0.12	2.51	0.03	0.25
		8Av	0.38	0.12	3.14	0.005	0.27
	L-ACC	d32	0.30	0.12	2.42	0.043	0.27
		a32pr	0.30	0.13	2.43	0.043	0.26
	R-ACC	25	0.37	0.12	3.07	0.006	0.27
		s32	0.36	0.12	2.93	0.01	0.26
GMV	L-DLPFC	p9.46v	0.51	0.12	4.27	<0.001	0.29
	R-ACC	s32	0.39	0.12	3.19	0.003	0.27

Abbreviations: SA=surface area, GMV=gray matter volume, DLPFC=dorsolateral prefrontal cortex, ACC=anterior cingulate cortex, L=left, R=right. R^**2**^=marginal R-squared. Full model results are provided in the supplementary material (Tables ST1–ST3).

## Data Availability

The data supporting this study are not publicly available due to their sensitive nature and privacy considerations.
