# Supplementary material for: Antidepressant Effects of Intermittent Theta-Burst Stimulation are Associated with Lower Baseline Regional Gray Matter Volumes and Surface Areas in Prefrontal Cortex
Source: J Psychiatr Res. Author manuscript; Available in PMC 2025 Dec 3. (PMC7618424; doi:10.1016/j.jpsychires.2025.11.006)
Supplement: Supplementary Material [file EMS211097-supplement-Supplementary_Material.docx]

**Supplementary Material**

**Table S1. All interactions between time, symptom improvement and GMV.**

| Variable | ROI | Coefficient | SE | Statistic | p-FDR |
| --- | --- | --- | --- | --- | --- |
| GMV | lh_L_25 | -0.0665434372221295 | 0.125190493483556 | -0.531537462394222 | 0.907116668263722 |
|  | lh_L_33pr | -0.0670755009383185 | 0.125189348501072 | -0.535792395610593 | 0.907116668263722 |
|  | lh_L_46 | 0.158644596481572 | 0.124856038360834 | 1.27062013631323 | 0.503312247197542 |
|  | lh_L_8Ad | 0.0388152486501392 | 0.125237532393265 | 0.309933036114551 | 0.956388286002904 |
|  | lh_L_8Av | 0.172504776739478 | 0.124781902155645 | 1.38245028934009 | 0.432198423086081 |
|  | lh_L_8BL | 0.178277705784251 | 0.124749178783113 | 1.42908921343844 | 0.408702516272143 |
|  | lh_L_8C | 0.249369991991864 | 0.124256859243004 | 2.00689115684296 | 0.130730959095259 |
|  | lh_L_9.46d | 0.111913038156447 | 0.12506003555108 | 0.894874510976268 | 0.681884678051153 |
|  | lh_L_9a | 0.0200335311319997 | 0.125255324442614 | 0.159941553152722 | 0.975897146957393 |
|  | lh_L_9p | 0.130894755472523 | 0.124985711900347 | 1.04727775265133 | 0.640943117496571 |
|  | lh_L_a24 | -0.151183801779181 | 0.124893359312295 | -1.21050312531947 | 0.536431395193932 |
|  | lh_L_a24pr | -0.175881685958759 | 0.124762892215303 | -1.40972754667502 | 0.418576479868025 |
|  | lh_L_a32pr | 0.245096923445467 | 0.12429113743848 | 1.97195816609839 | 0.140401815049572 |
|  | lh_L_a9.46v | -0.000396607497361204 | 0.125261781815154 | -0.00316622908930409 | 0.997476264295934 |
|  | lh_L_d32 | 0.178085889688501 | 0.124750283549419 | 1.42753895719968 | 0.408702516272143 |
|  | lh_L_i6.8 | -0.131921411142176 | 0.124981359249259 | -1.05552869591597 | 0.637550777468245 |
|  | lh_L_p24 | -0.102101205869838 | 0.125093883830863 | -0.816196625631091 | 0.712780826230802 |
|  | lh_L_p24pr | -0.0208333527442653 | 0.125254798309509 | -0.166327781653405 | 0.975897146957393 |
|  | lh_L_p32 | 0.159156868764106 | 0.124853409702816 | 1.27474987782025 | 0.503312247197542 |
|  | lh_L_p32pr | 0.115314190951544 | 0.125047575724047 | 0.922162547205371 | 0.668827374290339 |
|  | lh_L_p9.46v | 0.516971049967465 | 0.120883651293647 | 4.27660022207351 | 8.04662142932034e-05 |
|  | lh_L_s32 | -0.0654829880447061 | 0.125192748231346 | -0.52305735731353 | 0.907116668263722 |
|  | lh_L_s6.8 | 0.034460137266909 | 0.12524266967054 | 0.275146939597813 | 0.963320896018704 |
|  | lh_L_SFL | 0.0447503074800631 | 0.125229547873949 | 0.357346235291906 | 0.956388286002904 |
|  | rh_R_25 | 0.0888032518089534 | 0.125134791658717 | 0.709660763659948 | 0.792134424701234 |
|  | rh_R_33pr | -0.0241290094798387 | 0.125252413161862 | -0.192643070666089 | 0.975897146957393 |
|  | rh_R_46 | 0.162631697574258 | 0.124835352389127 | 1.3027695637636 | 0.489756208642763 |
|  | rh_R_8Ad | -0.0379569056241983 | 0.125238593185991 | -0.303076748617167 | 0.956388286002904 |
|  | rh_R_8Av | 0.27825663377896 | 0.12400931402393 | 2.24383656960851 | 0.0740903137116246 |
|  | rh_R_8BL | 0.0480498697220275 | 0.125224618079711 | 0.383709453131984 | 0.948844170157175 |
|  | rh_R_8C | 0.128640071228094 | 0.124995151047414 | 1.0291604926282 | 0.65137931579161 |
|  | rh_R_9.46d | 0.00756341128915256 | 0.125260863598643 | 0.0603812800891029 | 0.979097757096736 |
|  | rh_R_9a | -0.00333790715235964 | 0.125261605271035 | -0.0266474882318269 | 0.994940182626489 |
|  | rh_R_9p | -0.0281456715298534 | 0.125249033041575 | -0.224717675229563 | 0.975897146957393 |
|  | rh_R_a24 | -0.0565118281701944 | 0.125210371997477 | -0.451335039331513 | 0.948844170157175 |
|  | rh_R_a24pr | 0.0246629711474123 | 0.12525199403126 | 0.196906814443665 | 0.975897146957393 |
|  | rh_R_a32pr | 0.287891656322045 | 0.123920598178181 | 2.3231945338748 | 0.0610273474343932 |
|  | rh_R_a9.46v | 0.204460608346188 | 0.124587115937379 | 1.64110555740736 | 0.282580813003593 |
|  | rh_R_d32 | 0.0513376459352923 | 0.125219356934653 | 0.409981708835029 | 0.948844170157175 |
|  | rh_R_i6.8 | 0.214608416938632 | 0.124518279114172 | 1.72350933907346 | 0.240585640108246 |
|  | rh_R_p24 | 0.0292734420307869 | 0.125247990962971 | 0.233723845035099 | 0.974410582717511 |
|  | rh_R_p24pr | 0.0168438899520809 | 0.125257217757514 | 0.134474405975463 | 0.975897146957393 |
|  | rh_R_p32 | 0.116558504193745 | 0.125042923874885 | 0.932147942336751 | 0.668827374290339 |
|  | rh_R_p32pr | 0.0714808474215658 | 0.125179518108178 | 0.571026702305987 | 0.894686352126382 |
|  | rh_R_p9.46v | 0.126792503112746 | 0.125002762663564 | 1.0143176071556 | 0.65137931579161 |
|  | rh_R_s32 | 0.391902875666767 | 0.122764851538584 | 3.19230521403428 | 0.00468368247573413 |
|  | rh_R_s6.8 | 0.198492821060081 | 0.124626024821457 | 1.59270763345336 | 0.305659096086849 |
|  | rh_R_SFL | 0.015367546620132 | 0.12525798319929 | 0.122687163146174 | 0.976074254369042 |

**Table S2. All interactions between time, symptom improvement and SA.**

| Variable | ROI | Coefficient | SE | Statistic | p-FDR |
| --- | --- | --- | --- | --- | --- |
| SA | lh_L_25 | 0.105038843812485 | 0.125084076045251 | 0.839745930365155 | 0.612357551371893 |
|  | lh_L_33pr | -0.00707191658605498 | 0.125260979391978 | -0.0564574588222316 | 0.999913552733739 |
|  | lh_L_46 | 0.376671607376962 | 0.122956950983412 | 3.06344297223 | 0.00692754696669847 |
|  | lh_L_8Ad | 0.243878543395109 | 0.124300800710446 | 1.96200299596793 | 0.125244352983435 |
|  | lh_L_8Av | 0.286119201182305 | 0.123937149678101 | 2.30858303523549 | 0.0581060286638856 |
|  | lh_L_8BL | 0.207129451642483 | 0.12456933907785 | 1.66276431404229 | 0.224923145459621 |
|  | lh_L_8C | 0.448189555971323 | 0.12198581605941 | 3.67411204392029 | 0.000859435578312329 |
|  | lh_L_9 46d | 0.260407290903968 | 0.124165531935195 | 2.09725909312643 | 0.0934317022783744 |
|  | lh_L_9a | 0.111248861537755 | 0.125062424983059 | 0.889546652824177 | 0.583116583596949 |
|  | lh_L_9p | 0.246652376855135 | 0.124278729287613 | 1.98467089476202 | 0.119875270897941 |
|  | lh_L_a24 | 0.0434507246516921 | 0.125231393243175 | 0.346963517105646 | 0.958570472516604 |
|  | lh_L_a24pr | -0.115943452718978 | 0.125045229829664 | -0.927212120581615 | 0.555210605526358 |
|  | lh_L_a32pr | 0.301002212547774 | 0.123794921413196 | 2.43145848885922 | 0.0430703800366072 |
|  | lh_L_a9 46v | 0.306034963637497 | 0.123745156596324 | 2.47310660114021 | 0.0389505060243974 |
|  | lh_L_d32 | 0.300703746049403 | 0.123797844531959 | 2.42899015880503 | 0.0430703800366072 |
|  | lh_L_i6 8 | 0.109004315850816 | 0.125070394424812 | 0.871543712259945 | 0.595045591012348 |
|  | lh_L_p24 | 0.102606271216198 | 0.125092217274228 | 0.820245043632608 | 0.622538531845697 |
|  | lh_L_p24pr | 0.141565720391832 | 0.12493880390389 | 1.13308048395223 | 0.462792297183013 |
|  | lh_L_p32 | 0.237840069614553 | 0.124347972438776 | 1.91269760937723 | 0.137800999040998 |
|  | lh_L_p32pr | 0.174785857645162 | 0.124769101732236 | 1.40087453719324 | 0.344121060485183 |
|  | lh_L_p9 46v | 0.492616403027193 | 0.121293045117085 | 4.06137386155709 | 0.000194392054968825 |
|  | lh_L_s32 | 0.169373862781949 | 0.124799195489333 | 1.35717111090212 | 0.359409099019616 |
|  | lh_L_s6 8 | 0.136730388224141 | 0.124960516775147 | 1.09418872258805 | 0.479884473618254 |
|  | lh_L_SFL | 0.153096330258666 | 0.124883964535465 | 1.22590863309108 | 0.429088316389958 |
|  | rh_R_25 | 0.377589400192743 | 0.122945599562592 | 3.0711908481158 | 0.00682432394930446 |
|  | rh_R_33pr | 0.0374784235598822 | 0.125239174263223 | 0.299254796116041 | 0.977196299135848 |
|  | rh_R_46 | 0.26091568319117 | 0.124161228048649 | 2.1014264057451 | 0.0933139648284847 |
|  | rh_R_8Ad | 0.288784528415861 | 0.123912220455638 | 2.33055728768293 | 0.055391048988602 |
|  | rh_R_8Av | 0.386648343403747 | 0.122832016914525 | 3.14778144262503 | 0.00537161632242165 |
|  | rh_R_8BL | 0.237887990122635 | 0.124347602779641 | 1.91308867083029 | 0.137800999040998 |
|  | rh_R_8C | 0.282486990640114 | 0.123970742193271 | 2.27865854186559 | 0.0621925607434747 |
|  | rh_R_9 46d | 0.180400417262484 | 0.124736873204821 | 1.44624771030024 | 0.325854298449578 |
|  | rh_R_9a | 0.0818365710565809 | 0.125153944177151 | 0.653887271349149 | 0.754957155505843 |
|  | rh_R_9p | 0.138543835845637 | 0.124952462418834 | 1.10877235361113 | 0.474585968838697 |
|  | rh_R_a24 | 0.0474064393850196 | 0.125225606954418 | 0.378568254033502 | 0.936109320949544 |
|  | rh_R_a24pr | 0.0301146341718016 | 0.125247186769698 | 0.240441601512183 | 0.977196299135848 |
|  | rh_R_a32pr | 0.277104930119156 | 0.124019712566222 | 2.23436197669941 | 0.0683697246764936 |
|  | rh_R_a9 46v | 0.262288910813474 | 0.124149561321632 | 2.11268495853937 | 0.0916132997911046 |
|  | rh_R_d32 | 0.210413261853655 | 0.124547145575293 | 1.68942660935134 | 0.215330988736203 |
|  | rh_R_i6 8 | 0.310757852714967 | 0.123697686646198 | 2.5122365756427 | 0.0353224671222892 |
|  | rh_R_p24 | 0.0646060957349898 | 0.125194585294023 | 0.516045447039585 | 0.855928163399841 |
|  | rh_R_p24pr | 0.116788182430131 | 0.125042059819259 | 0.933991191435437 | 0.554920572899603 |
|  | rh_R_p32 | 0.21020965898448 | 0.124548531851231 | 1.687773078173 | 0.215330988736203 |
|  | rh_R_p32pr | 0.224691719045594 | 0.124446536969367 | 1.80552809678346 | 0.171859092778043 |
|  | rh_R_p9 46v | 0.254594053874227 | 0.124214133789008 | 2.04963836326939 | 0.103809402367466 |
|  | rh_R_s32 | 0.361446984065979 | 0.123141076287788 | 2.9352267737311 | 0.0102942399879388 |
|  | rh_R_s6 8 | 0.281452134761997 | 0.123980232561752 | 2.27013717385803 | 0.0629880249861187 |
|  | rh_R_SFL | 0.227292791634596 | 0.124427489174456 | 1.8267088176626 | 0.165469377017493 |

**Table S3. All interactions between time, symptom improvement and CT.**

| Variable | ROI | Coefficient | SE | Statistic | p-FDR |
| --- | --- | --- | --- | --- | --- |
| CT | lh_L_46 | -0.562548665520482 | 0.791828534489018 | -0.71044252766605 | 0.816041522059864 |
|  | lh_L_8Ad | 0.218422163948085 | 0.818108192124064 | 0.266984447840564 | 0.960428305792031 |
|  | lh_L_8Av | 0.0435563607810217 | 0.6011243851219 | 0.0724581498589333 | 0.987370656804555 |
|  | lh_L_8BL | 0.680033577035793 | 0.501824438689818 | 1.35512247831383 | 0.678037979847986 |
|  | lh_L_8C | 0.549697140548126 | 0.844420637416149 | 0.650975492771174 | 0.823771058501537 |
|  | lh_L_9.46d | 0.033817208172949 | 0.796531298444946 | 0.0424555924405856 | 0.994161666006496 |
|  | lh_L_9a | 0.866657533329897 | 0.743386532875138 | 1.16582355881266 | 0.678037979847986 |
|  | lh_L_9p | 0.136370606110019 | 0.751384886972397 | 0.18149234629872 | 0.97400006190692 |
|  | lh_L_a9.46v | -0.522269347521186 | 0.863473359073178 | -0.604847088834069 | 0.854348260175133 |
|  | lh_L_i6.8 | 0.275165444762623 | 0.703561382918986 | 0.391103678290297 | 0.94608700750512 |
|  | lh_L_p9.46v | 1.56799878939858 | 0.688358370977053 | 2.27788148660554 | 0.357501134635351 |
|  | lh_L_s6.8 | 0.910660451050944 | 0.696123599996958 | 1.30818787217518 | 0.678037979847986 |
|  | lh_L_SFL | 1.01852140490661 | 0.613636322322871 | 1.65981277159585 | 0.589296206360743 |
|  | rh_R_46 | 0.493866605604936 | 0.741216534453444 | 0.666291943917713 | 0.82176172808379 |
|  | rh_R_8Ad | -0.285968012452869 | 0.777402247886638 | -0.36785076609988 | 0.94608700750512 |
|  | rh_R_8Av | 0.706222122372663 | 0.631900655118534 | 1.11761574648184 | 0.678037979847986 |
|  | rh_R_8BL | 0.378895314839707 | 0.636793074745058 | 0.595005394792333 | 0.855301732522076 |
|  | rh_R_8C | -0.171093463762831 | 0.837661067523746 | -0.204251421483165 | 0.973421210686597 |
|  | rh_R_9.46d | 0.17340955773921 | 0.752941002462485 | 0.230309622097982 | 0.973421210686597 |
|  | rh_R_9a | 0.924421145925879 | 0.677444150643099 | 1.36457174373463 | 0.678037979847986 |
|  | rh_R_9p | 0.10285304800521 | 0.676559116362633 | 0.152023741189352 | 0.981688390926027 |
|  | rh_R_a9.46v | 0.366319714921623 | 0.773552888986139 | 0.473554840447616 | 0.944512292775063 |
|  | rh_R_i6.8 | 2.00684772265776 | 0.612033117477196 | 3.2789855080555 | 0.163002186165437 |
|  | rh_R_p9.46v | -0.0112559708090957 | 0.773535752888029 | -0.0145513258657677 | 0.994161666006496 |
|  | rh_R_s6.8 | 0.787145221971982 | 0.616151807543336 | 1.2775183198933 | 0.678037979847986 |
|  | rh_R_SFL | 0.17306017061048 | 0.577752459318819 | 0.299540344344913 | 0.957347162001232 |
|  | lh_L_25 | -0.20131501929721 | 0.33806658482811 | -0.595489256649156 | 0.855301732522076 |
|  | lh_L_s32 | 0.00716914015794414 | 0.446273733805196 | 0.0160644456863185 | 0.994161666006496 |
|  | rh_R_25 | -0.610227386174716 | 0.376221472400835 | -1.62198978777204 | 0.599018023292957 |
|  | rh_R_s32 | 0.523038986334543 | 0.548816350384667 | 0.953030983803496 | 0.698945291687681 |
|  | lh_L_a24 | -0.385532243567908 | 0.581425406968905 | -0.663081177648858 | 0.82176172808379 |
|  | rh_R_a24 | 0.951752665080496 | 0.674639389066962 | 1.41075762919326 | 0.678037979847986 |
|  | lh_L_p24 | -0.431540810948767 | 0.548237495146281 | -0.787142095842283 | 0.760480565858261 |
|  | rh_R_p24 | 0.466124891729288 | 0.43014651394725 | 1.08364214660694 | 0.678037979847986 |
|  | lh_L_p32 | 0.275890756770668 | 0.715348990739186 | 0.385672951723304 | 0.94608700750512 |
|  | rh_R_p32 | 0.445969014944063 | 0.551370725992785 | 0.808836947484039 | 0.750202796094753 |
|  | lh_L_d32 | 0.765446424136635 | 0.621811477747291 | 1.23099436329112 | 0.678037979847986 |
|  | rh_R_d32 | 0.105180087218108 | 0.65838138264721 | 0.159755561123556 | 0.981688390926027 |
|  | lh_L_a32pr | 0.886454964314514 | 0.665076247877239 | 1.33286216000626 | 0.678037979847986 |
|  | rh_R_a32pr | 1.25650556268329 | 0.658294506810212 | 1.90872861566434 | 0.424257191431542 |
|  | lh_L_a24pr | 0.0588840597261857 | 0.537715962806373 | 0.109507739771879 | 0.981688390926027 |
|  | rh_R_a24pr | 0.289789446508148 | 0.416657187557114 | 0.695510494387966 | 0.816089255184632 |
|  | lh_L_33pr | 0.0305835732553712 | 0.393439210120563 | 0.0777339229763078 | 0.987370656804555 |
|  | rh_R_33pr | 0.455007548396306 | 0.397002285723728 | 1.14610813277016 | 0.678037979847986 |
|  | lh_L_p32pr | 0.540161742830696 | 0.803450677958522 | 0.672302305106253 | 0.82176172808379 |
|  | rh_R_p32pr | 0.529090729645727 | 0.757326571939536 | 0.69862955988816 | 0.816089255184632 |
|  | lh_L_p24pr | -0.00174889323454666 | 0.574586154110477 | -0.0030437441313812 | 0.997573894213073 |
|  | rh_R_p24pr | 0.85005765738412 | 0.642454351094283 | 1.32314094524573 | 0.678037979847986 |

**Fig. S1. Modeling Parameters**


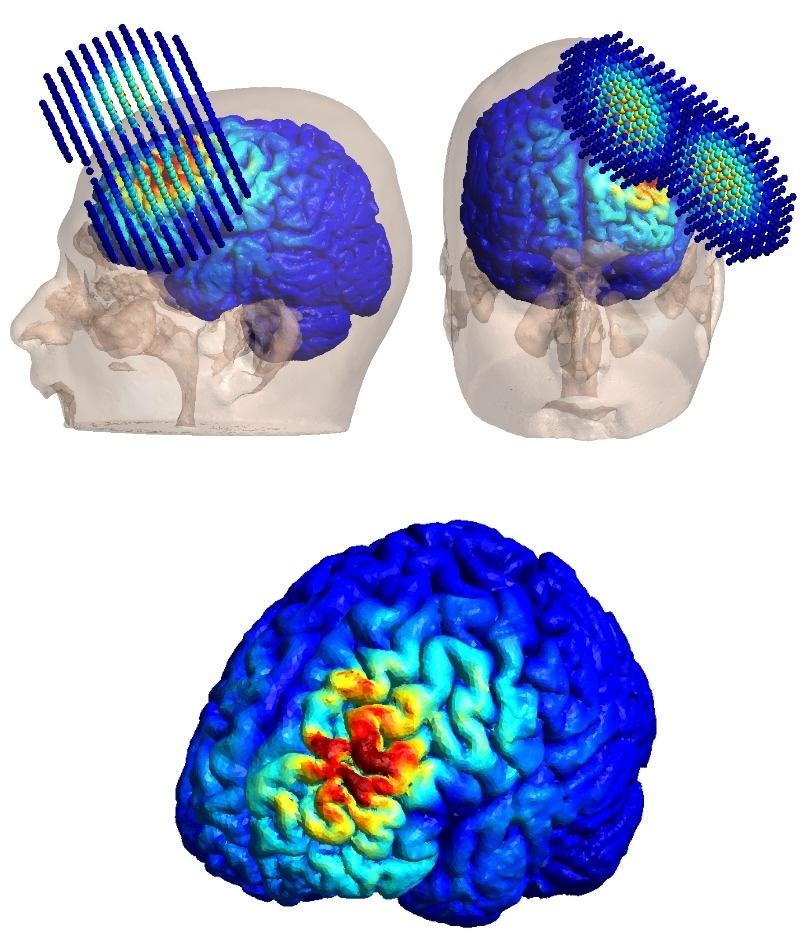


For the E-field analysis, we used SimNIBS version 4.0.0. The head models were created using CHARM routine based on individual T1- and T2-weighted structural resonance imaging, which allowed for more accurate segmentation. The tetrahedral head meshes resulting from the segmentation procedure were used to simulate the E-field distribution. For E-field modeling, the coil was simulated towards ‘Nz’ and centered in F3, with a distance of 1 cm from the scalp. The dl/dt values were configured based on the MT of each individual. Assuming that maximal machine output is 150 A/µs using a Cool B65 coil, we calculated the individual MT by multiplying 150 A/µs with the stimulator intensity related to 110% of the rMT. Thus, the dl/dt for an individual
